# Biofoundries and citizen science can accelerate disease surveillance and environmental monitoring

**DOI:** 10.3389/fbioe.2022.1110376

**Published:** 2023-01-12

**Authors:** Martin Holub, Ethan Agena

**Affiliations:** ^1^ Department of Bionanoscience, Delft University of Technology, Delft, Netherlands; ^2^ Department of Chemical Engineering and Applied Chemistry, University of Toronto, Toronto, ON, Canada

**Keywords:** biofoundry, biosecurity, biosurveillance, citizen science, policy

## Abstract

A biofoundry is a highly automated facility for processing of biological samples. In that capacity it has a major role in accelerating innovation and product development in engineering biology by implementing design, build, test and learn (DBTL) cycles. Biofoundries bring public and private stakeholders together to share resources, develop standards and forge collaborations on national and international levels. In this paper we argue for expanding the scope of applications for biofoundries towards roles in biosurveillance and biosecurity. Reviewing literature on these topics, we conclude that this could be achieved in multiple ways including developing measurement standards and protocols, engaging citizens in data collection, closer collaborations with biorefineries, and processing of samples. Here we provide an overview of these roles that despite their potential utility have not yet been commonly considered by policymakers and funding agencies and identify roadblocks to their realization. This document should prove useful to policymakers and other stakeholders who wish to strengthen biosecurity programs in ways that synergize with bioeconomy.

## Introduction

Humans have always been at prey to natural pathogens. There have been at least fifteen epidemics with a death toll over 1 million in the last 500 years (one every 33 years on average). Two occurrences of bubonic plague, a bacterial respiratory infection, in the 6th and 14th century wiped out an estimated half of the worldwide population. Spanish flu, a viral respiratory infection, caused tens of millions of deaths in the early 20th century. More recently, the coronavirus pandemic caused millions of deaths worldwide. While the most shocking due to their rapid development, pandemics are only one of major global health risks. Another global health risk is due to antibiotic resistance. Increasingly prevalent among pathogens, it is causing an increase in the number of deaths due to bacterial infections globally (Zhang et al., 2022). Furthermore, as we become increasingly able to edit and engineer living organisms, man-made pathogens could be at the source of future health threats as well. Driven to protect ourselves from the often-lethal forces of nature, we as humans have learnt to shape our environments in many ways early on. From building shelters to growing crops, these efforts have paid out wildly, testified by how well we have done as a species. It has been only very recently, however, that we are developing more appreciation for how we have influenced and continue to influence the natural environment around us in this process. Environmental pollution, climate change and biodiversity loss are just some examples. One of the less known consequences is an emergence of novel urban ecosystems that give rise to novel species (Danko et al., 2021). Risks to the health of humans and our environment must be monitored, as any attempts to manage and contain them in the future will have to rely on data to be effective. Biosurveillance (detection of biological threats to human health) and environmental monitoring (observation and characterization of the natural environment) are both processes of provisioning this data. In the recent case of coronavirus pandemic, biosurveillance through routine testing and contact tracing on the level of individuals has proved to be crucial to the coronavirus pandemic response worldwide. Additionally, aggregate monitoring of coronavirus through wastewater sampling has proved to be a predictive signal to case counts and hospital load independent of direct diagnostic data (Venugopal et al., 2020; Zhu et al., 2021; Calderón-Franco et al., 2022; Calderón-Franco et al., 2021). In a similar fashion, the benefits of biological monitoring have been seen for targets other than infectious disease such as tracking of bacterial antibiotic resistance in the environment (Huijbers et al., 2019), and even conservation efforts through the analysis of environmental DNA (Francis Thomsen and Willerslev, 2015).

### Current bottlenecks in biological monitoring

Despite some successes, biological monitoring programs generally fall short on a multitude of levels when it comes to preparedness for detection and prevention of future biological risks. While, to our knowledge, there is no resource comprehensively reviewing and comparing biosecurity programs across the world, Nuclear Threat Initiative (NTI) has compared 195 countries in terms of preparedness for pandemics and epidemics in Global Health Security Index (www.ghsindex.org). The United States ranked number one in 2021 and this, together with its being relatively well researched in academic literature, is one of the main reasons why we use it as an illustrative example. It is likely that the US system is average or above-average compared to biodefense systems across the world and that its shortcomings will reflect common shortcomings worldwide.

**FIGURE 1:**
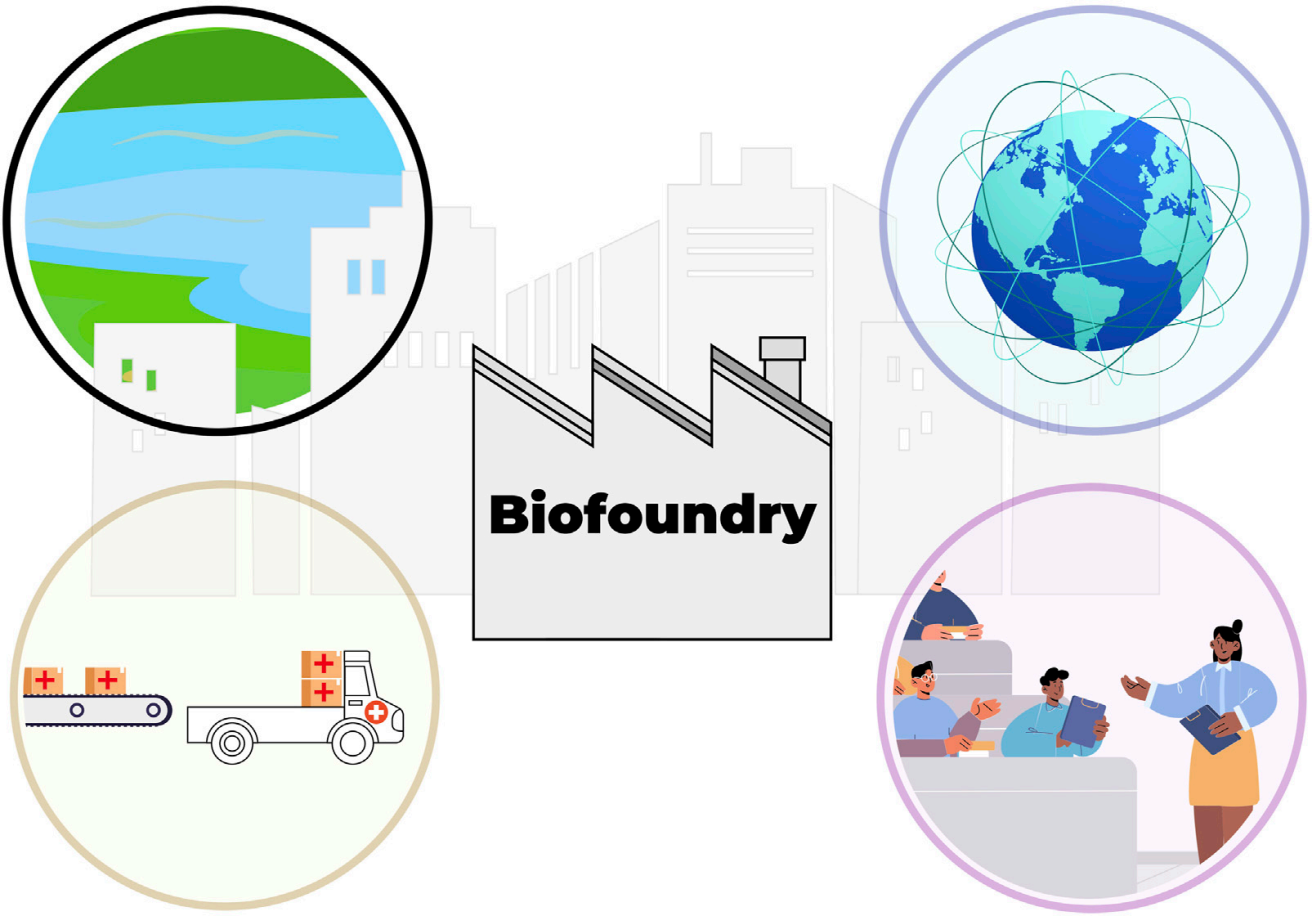
A vision for the future role of biofoundries and citizen science. Biofoundries are local hubs that are close to urban areas, and foster citizen engagement through citizen science (top left) and education (bottom right). Global network of biofoundries cooperates to share protocols and data (top right), which further strengthens the capacity of individual biofoundries to safeguard biosecurity and implement interventions.

The main shortcomings of the US biodefense as reviewed by the Bipartisan Commission on Biodefense (Bipartisan Commission on Biodefense, 2022) are lack of quick response capability (Vickers and Freemont, 2022) and general lack of structured investment, lack of adequate data interoperability and data collection standards, and poorly developed regulatory structure. Several biosurveillance bottlenecks, such as insufficient testing and processing capacity, where at one point a single facility was responsible for handling samples nation-wide, became manifest during the COVID-19 pandemic and limited the speed of delivering public health interventions (Boeckh et al., 2022). This ultimately encouraged establishment of more distributed testing sites and accessing unconventional sequencing facilities for diagnostic work, such as academic laboratories (Kim et al., 2020). Coupled with the increased public awareness of biosecurity as result of the pandemic, along with the identification of bottlenecks in current biosurveillance programs, the question arises: Is there a different way to structure biosurveillance programs that could improve outcomes? In this paper we argue for options to do so by considering the newly emerging infrastructure of highly automated facilities for processing of biological samples, biofoundries (Box 1; Figure 1). In the following sections we discuss how this infrastructure can be exploited to benefit not only response to disease outbreaks, but also the response to more subtle targets in health, ecology, and biosecurity. We identify several opportunities at this interface, most of which have not been commonly considered by policymakers and funding agencies. These include developing measurement standards and protocols, engaging citizens in data collection, decentralized manufacturing (Box 1), and processing of samples. We then finish by highlighting roadblocks to their realization. In this vision we focus on biofoundries that are run and funded by the public sector. While industry-owned biofoundries exist and undoubtedly deliver value, they may be subject to unique agendas of their owners and we do not see them as a suitable foundation of national biosecurity. In contrast, we believe that less-formal infrastructure for biological experimentation, such as bio-hack spaces and bio-DIY labs, can contribute to these ends in various ways, including increasing the impact of citizen scientists, as well as encouraging safe practices, through collaboration with biofoundries and community engagement. However, due to specific challenges these spaces currently face, including lack of appropriate regulatory schemes, issues with securing suitable lab space and equipment, as well as negative sentiment among broad public, we anticipate that their contribution will develop only as they mature on medium and long term. We therefore leave them out of scope of the present discussion and refer interested reader to recent reviews on the topic (Seyfried et al., 2014; Meyer., 2013; Landrain et al., 2013; Keulartz and van den Belt, 2016).

## Standardized and automated measurement workflows facilitate biosurveillance

The cornerstone of biofoundry operations is the melding of automation of standardized bioengineering workflows and the design, build, test and learn cycles. Without these principles implemented, the difference in throughput achieved by biofoundries as compared to typical laboratories would not be possible (see Box 1 for a general introduction to biofoundries). The outcomes of engineering biology can be variable due to the complexity of biological systems and the magnitude of unknowns and confounding factors. Thus, by leveraging automation technologies throughout sampling, processing, and analysis, as much human variability is removed from the process which allows for gains in consistency of results while shortening the timescale of workflows. This approach is also suitable for processing many samples at the same time, allowing to explore unprecedented breadth of genetic variability. While mainly employed for sample processing, experimentation, and analysis by academics and researchers, biofoundries are also well suited to boost our ability to rapidly collect and analyze samples originating from patients, or the environment. In a recent example, Ginkgo Bioworks has used its high-throughput sequencing capabilities to support nation wide efforts in COVID-19 testing, as well as supported vaccine manufacturers in optimizing their products (Cho, 2020). On a similar note, automatized routines adopted at biofoundries, as well as their equipment, make them good candidates for handling samples with pathogenic potential. Aside from automated processing of high numbers of samples, biofoundries are particularly suited for development of measurement standards and standardized calibration samples. Their nature as a collaborative platform, that can interface with governmental entities, further facilitates encouragement and adoption of so developed standards (Mao et al., 2021). In the context of biosurveillance, adoption of these standards enables comparison of results across time and geographical regions and enables their users to harmonize interventions. An example is provided by London Biofoundry, which developed a rapid automated SARS-CoV-2 testing platform that was deployed and scaled in national diagnostic labs and could be also adopted by other biofoundries (Crone et al., 2020).

BOX 1 **Tools for Rapid and Robust Biological Surveillance**

**Biofoundries**

**FIGURE 2:**
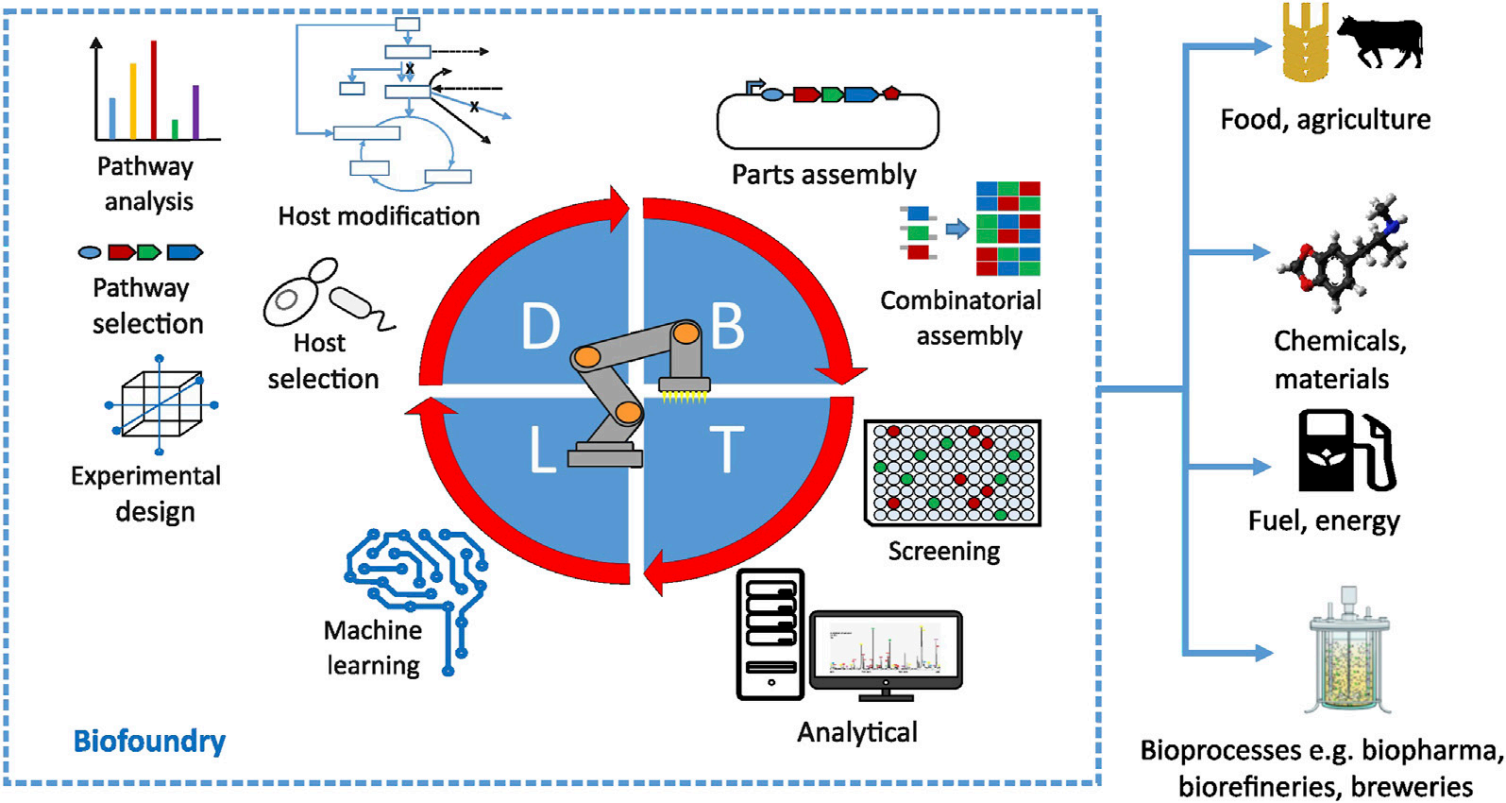
Overview of major processes in a biofoundry happening at design (D), build (B), test (T) and learn (L) stages of the development cycle. Reprinted with permission from ref. (Philp, 2021).

A biofoundry is a highly automated facility for processing of biological samples. In that capacity it has a major role in accelerating innovation and product development in engineering biology by implementing design, build, test and learn (DBTL) cycles (Hillson et al., 2019) (Figure 2). The equipment in biofoundries typically include automated liquid handling systems, high-throughput sequencing and chemical analysis equipment, and a software ecosystem for data and personnel management (Hillson et al., 2019). For example, one of the largest biofoundries and synthetic biology companies in operation today, Ginkgo Bioworks, has leveraged their integrated system of automated bioengineering to evaluate on the order of tens of thousands of engineered strains (Ginkgobioworks, 2022) — a quantity that can not be achieved with bench-scale workflows alone. Dropping costs of DNA synthesis and sequencing, development of facile technologies for genome editing, lab-on-chip microfluidics, and expanding ecosystem of hardware and software automation tools are some of the main factors that contribute to synthetic biology as an engineering discipline. The growth of bioeconomy enabled by these technological advances goes hand in hand with the increasing popularity of biofoundries. The establishment of the Global Biofoundry Alliance (GBA), which has grown to over 30 members since 2019 (Hillson et al., 2019), including 14 biofoundries in Australia and Asia, nine in North America and 10 in Europe, is a sign of the continued growth of this sector. Importantly, first steps towards establishment of biofoundries in Latin America (The Bridge Biofoundary, 2022) and Africa (Thimiri Govindaraj, 2022) are already underway. Aside from their direct role in biological experimentation, biofoundries serve as platforms that bring public and private stakeholders together to share resources, develop standards and forge collaborations on national and international level (Vickers and Freemont, 2022). In that capacity they can gather sufficient momentum to realize collaborative projects that may need top-down incentive or broader consensus for economical viability (e.g., projects contributing to environmental sustainability), contribute to development of legal and ethical frameworks by shaping governance of emerging fields (Mao et al., 2021) and manage the relationship with the public. Despite their obvious utility, the high establishment, personnel and overall running costs make the business case for biofoundries difficult. While there is early evidence that biofoundries deliver high added value through innovation and knowledge creation (Winickoff et al., 2021), it is useful to consider additional roles for biofoundries that could strengthen their business case, which could further rationalize their establishment in countries with lower research budgets.

**Citizen science**

Citizen science, which is the involvement of the public in scientific research, can range from collecting and analyzing data to prototyping low-cost sensing devices. Digitalization of our society and adoption of open-data and open-innovation paradigms are the main contributors to its rise in recent two decades (Maccani et al., 2020). The main benefits of citizen science are two-fold: 1) citizen science contributes to and expands research, and 2) it shapes the relationship between scientists and the public in an engaging, two-way interaction (Hecker et al., 2018; Den Broeder et al., 2016). The first benefit enables a larger breadth of research than what is achievable by an academic laboratory alone, e.g. collection of data at higher spatial resolution, or making measurements of completely new parameters. The latter allows citizens to familiarize themselves with the scientific method and gain insight on interpretability and accuracy of collected data, as well as reciprocally provide feedback on collected data and the process of its acquisition. Recent incorporation of citizen science concepts into university (MOOC, 2022; UZH, 2022) and high-school (Developer Community, 2018) curricula suggest that its impact will continue to rise.

**Cell-free synthetic biology**

Standardization could be facilitated by adoption of cell-free systems (CFS). CFS could also contribute to a shift towards decentralization of manufacturing. Cell-free gene expression is gaining popularity in synthetic biology and bioengineering (Garenne et al., 2021). Diverse applications including protein production, therapeutics manufacturing and biosensing all can benefit from by-passing living cells. Benefits include facilitated rapid prototyping and condition screening, reduction of reaction volumes, higher predictability and amenability to mathematical modeling. Consequently, cell-free biomanufacturing is of imminent interest also beyond academia. Furthermore, engineered cell-free systems are not classified as genetically engineered organisms (Khambhati et al., 2019; Taylor and Wieden, 2021), which simplifies biosafety and biosecurity of their application. Adoption of cell-free systems further decreases batch-to-batch variability (Kumar Dondapati et al., 2020), reduces sample volumes and lowers regulatory barriers. Biofoundries are particularly suited to drive the transition to decentralized biomanufacturing through adoption of cell-free systems. The integrated design, build, test and learn cycle, the automation facilities for liquid handling, and the standardization in biofoundries are all vital to rapid, scalable and reproducible processes. Geographical distribution of biofoundries allows them to serve as local hubs (Hillson et al., 2019), out of which products based on CFSs can be rapidly deployed, for instance in the case of response to health and environmental crises.

## Biosurveillance enabled by biofoundries and citizen scientists

Areas that can benefit from citizen science (Box 1) are diverse. With an aging population and increasing obesity rates on one hand, and ongoing prevalence of malnutrition, in both developed and developing countries, on the other (Jain et al., 2021), public health monitoring emerged as an important area for application of citizen science. In The American Gut project (The Microsetta Initiative, 2022) scientists receive stool samples from the public with the aim of identifying the relationships between health and lifestyle and the microbiome. The 100 For Parkinson’s project (The Parkinson’s Blog, 2022) invited people across the United Kingdom and United States to track their health for 100 days with a mobile app, with the aim of understanding how technology can support Parkinson patients. The Seattle Flu Study (Seattle Flu Alliance, 2021) focuses on studying seasonal influenza, aiming to understand how it develops and spreads in the Seattle area. Participants are typically asked to regularly answer simple survey questions and if they are identified as high-risk, they are sent a testing kit and asked to submit the swab back by post or to report the result of a self-test. Thanks to the high number and broad distribution of samples, The Seattle Flu Study was among the first to discover and identify COVID-19 in the Seattle area (Chu et al., 2020), clearly highlighting the utility of citizen science in public health monitoring and protection. Overall, these examples demonstrate the utility of citizen science programs outside of conventional academic and medical studies on assessing healthcare outcomes and impacts.

Synthesizing the capabilities of biofoundry facilities with the breadth of sampling possible with citizen-based science programs described above brings a new conception for biological monitoring and surveillance to light. When considering the limitations of citizen science programs, in terms of the input variability and the magnitude of samples collected, leveraging the processing pipeline of a biofoundry may allow more consistent results to be obtained. Furthermore, biofoundries could act as formal knowledge hubs which if engaged appropriately with the local community could facilitate the quality of input from citizen scientists. Both these aspects could encourage the establishment of more citizen science programs as biofoundries can effectively reduce some of the technical hurdles associated with citizen science. As another consideration, the automation technologies leveraged in biofoundries also enable the incorporation of additional engineering controls in the handling of hazardous samples that could de-risk many hazardous biosurveillance targets. Overall, the synergies between biofoundry automation and standardization, and the collaborative nature of biofoundries as interface between public and private sectors are all factors that point to utility and feasibility of expanding the applications of citizen science to more elusive biosurveillance targets that could strengthen existing biodefense programs and could have positive impacts on our ability to monitor the environment and public health.

## Biosecurity-related activities are source of funding and direction of development for biofoundries

Biofoundries are useful to the communities of their users as hubs with dedicated instrumentation and support of skilled staff. Sample handling and processing can be automated and standardized, carried out at small and medium scale rapidly and reproducibly. Resulting data are appropriately stored and processed, often in cooperation with trained bioinformaticians. However, acquiring and maintaining dedicated equipment carries cost. Equally importantly, salaries of highly-skilled employees, together with costs for consumables for experiments, contribute to high running costs of a biofoundry (Holowko et al., 2021). Consequently, putting together a viable business model for biofoundry is challenging. Above we have outlined how biofoundries can foster and support biosecurity, bio- and environmental-surveillance efforts by various means including standardization of samples and protocols, engagement with citizen scientists, and interface with decentralized manufacturing facilities. We believe that these further strengthen rationale for structural public investment into biofoundries and that national security agencies, environmental protection agencies, and related institutions can reap substantial benefits from channeling some of their financial resources into biofoundry operations. Aside from enabling biosurveillance, such effort contributes to training of staff at the forefront of biological engineering and biorisk and environmental monitoring, which is a valuable asset for national economy and security both long and short term. Furthermore, such trained staff, at the disposition of biofoundry infrastructure, will be instrumental to establishment of biosecurity training programs for professionals across the fields of security, intelligence and law reinforcement. This was recently exemplified by hands-on introductory to synthetic biology developed in collaboration between the Federal Bureau of Investigation (FBI) and the Colorado State University (CSU) (Neil et al., 2019). Recent years have seen growing interest in and implementation of decentralized biomanufacturing facilities (also called biorefineries) (Kritharis et al., 2022). While decentralized manufacturing will likely develop infrastructure separate from biofoundries, there is potential for their synergy in bioeconomy as well as in health and biosecurity targets. Biofoundry-enabled surveillance would likely lead to shorter feedback cycles, earlier risk detection and ability to respond more locally to potential outbreaks. Such response could be further sped up by access to localized biomanufacturing facilities that would have the ability to develop therapeutic or other responses. Similarly as the ability to produce crops locally contributes to food supply chain security and sustainability, so will decentralized biomanufacturing contribute to local security and sustainability. The rise of the bioeconomy suggests that this contribution will play out on multitude of levels including therapeutics, materials, fuels and food.

## Conclusion

Biological risks, including pandemics or rapid rise of antimicrobial resistance, are commonly regarded as potentially existential to humanity (FHI, 2022; CSER, 2021). Even if not fatal, biological catastrophes and engineered attacks have the potential to significantly impact lives of many, spreading rapidly to large geographical areas. Biosecurity therefore should be a critical priority for national security agencies (NSAs) worldwide. Similarly, climate change leads to gradual change of environmental conditions impacting ecosystems globally, also imposing existential threats to humanity. Accurate, wide-spread and time-resolved monitoring is crucial for effective interventions and policy making in these scenarios. Biosurveillance at the required level of spatial and temporal resolution remains challenging. Required number of samples and collection points is usually high. Moreover, samples may be perishable or pathogenic, complicating transport. In this paper, we have argued that biofoundry facilities can support several ways to improve our ability to carry out biosurveillance. They can function as distributed hubs of data collection and analysis, empowering biosurveillance by reducing transport times. Their distributed nature further confers the system with robustness, e.g., in case of a targeted attack. They can play a key role in developing standard protocols and standardized samples and work with citizens to develop new sample collection schemes. Finally, they can collaborate with biorefineries for small scale rapid production of therapeutic compounds.

**FIGURE 3:**
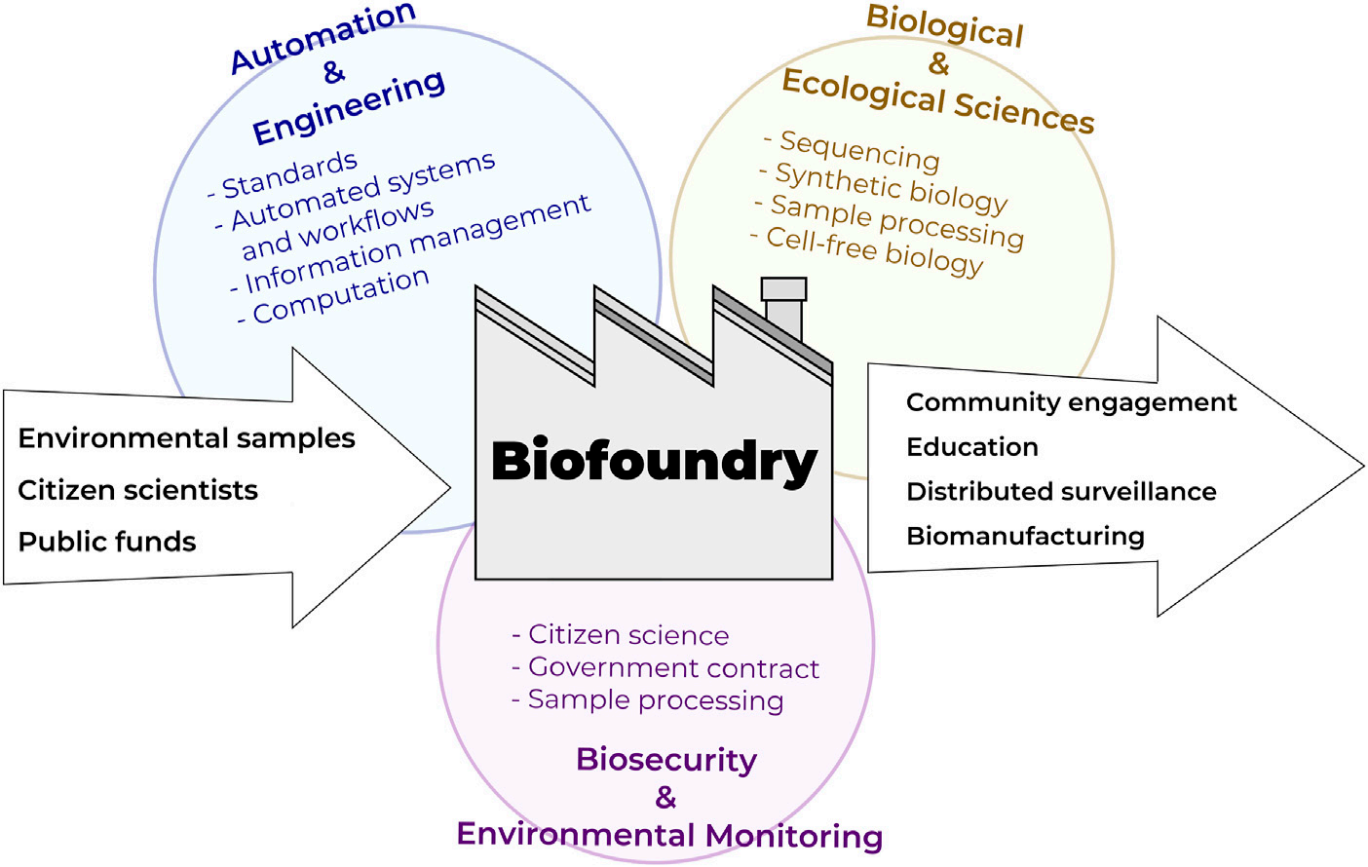
Biofoundries at the nexus of automation technologies, bioengineering, and biological/ecological monitoring interfacing with citizen science programs.

While there is potential for the vision presented in this paper (Figure 3), biofoundries worldwide are still in their early stages of development and such biosurveillance programs have challenges barring implementation. We have identified some key barriers, as well as some directions to address these below.

• **Develop biosecurity policy to leverage biofoundries.** Foremost, biofoundries may not be eligible for biosurveillance related operations and or funding as they may not qualify for the correct biosafety clearance in their jurisdiction. Regulatory frameworks and granting programs, which differ jurisdiction to jurisdiction, should be reviewed with biofoundries in mind so that appropriate amendments, that support the biosecurity capacity of biofoundries, can be identified. Additionally, with the continued creation of biofoundries worldwide, it is imperative that a unified development of standards be created and adopted such that the benefit of standardization can be preserved between nations.
• **Design biofoundries with sufficient biosafety level.** Bifoundries are currently mostly designed and classified at the biosafety level 1. In order to be able to use their capacities for broad-spectrum pathogen monitoring, they will have to classify for biosecurity level 2 clearance. There is a need for collaboration between biofoundries and biosafety regulators to apply and adapt the regulations to biofoundry use cases.
• **Expand use cases for biofoundries to include citizen science.** Citizen science programs may not be currently considered as a part of a biofoundry’s use cases. Thus, a biofoundry’s engagement with citizens and citizen science groups may not be adequate and could preclude their use by these groups. Therefore, it is recommended that established, and up and coming biofoundries, ensure that citizens and citizen science groups are included in the development of their facilities and invited to participate in biofoundry operations.
• **Create incentives to encourage biofoundry establishment.** As biofoundries are at the confluence of automation and biological technologies, they have the potential to closely cooperate with decentralized biomanufacturing facilities, and catalyze their further emergence. With the increasing growth in this sector, incentives for the establishment of biofoundries should be put forth as it could not only enable efforts in engineering biology, but could also help drive the transition to a circular bioeconomy.
• **Equip future biologists with quantitative and engineering skills.** While many universities have adapted their study programs and include increasing amounts of quantitative, programming and even hardware skills in their curricula, these efforts require broader adoption to build a future workforce that can effectively work at the nexus of technology and biology and continue to push it forward. As biofoundry operations and related facilities become more common, the need for such skills will continue to rise.

Biofoundries are growing in prevalence year over year, and this growth highlights the importance of assessing the role biofoundries can play in a nation’s biosecurity program. Synergies with citizen science could potentially extend the breadth of biosurveillance to more subtle targets than before by leveraging biofoundry facilities. Should the concepts in this paper be implemented, it could have transformative impacts on the way we monitor health, ecology, and biosecurity, by distributing the load among a network of biofoundries.

## Data Availability

The original contributions presented in the study are included in the article/supplementary material, further inquiries can be directed to the corresponding author.
